# Mediator Complex Subunit 19 Promotes the Development of Hepatocellular Carcinoma by Regulating the AKT/mTOR Signaling Pathway

**DOI:** 10.3389/fonc.2021.792285

**Published:** 2022-01-03

**Authors:** Yuting Zhang, Peifang Qin, Xingfeng Xu, Mao Li, Haitao Huang, Jianguo Yan, Yali Zhou

**Affiliations:** ^1^ Department of Microbiology, Guilin Medical University, Guilin, China; ^2^ Key Laboratory of Tumor Immunology and Microenvironmental Regulation, Guilin Medical University, Guilin, China; ^3^ Department of Physiology, Guilin Medical University, Guilin, China

**Keywords:** hepatocellular carcinoma, *MED19*, *AKT/mTOR* signaling pathway, proliferation/migration/invasion, tumor immune infiltration

## Abstract

**Background:**

Hepatocellular carcinoma (HCC) is one of the most common malignant tumors, the pathogenesis of which remains unclear. Mediator complex subunit 19 (*MED19*), a subunit of the Mediator complex, is a multi-protein co-activator necessary for DNA transcription factors to induce RNA polymerase II transcription. In the current study, we aimed to study the role of *MED19* in HCC and elucidate its mechanism.

**Methods:**

*MED19* expression in HCC tissues was determined. The relationship between *MED19* and the clinical prognosis was explored. The influence of *MED19* on HCC cell viability, migration, invasion, and apoptosis was studied. The expression of *AKT/mTOR* pathway genes and proteins was detected by qRT-PCR and western blot. The correlation between *MED19* and immune infiltration was investigated.

**Results:**

*MED19* was upregulated in HCC tissues compared with tumor-adjacent tissues, and was associated with a poor prognosis. Furthermore, high *MED19* expression was correlated with race, gender, *etc.* Knockdown of *MED19* inhibited cell proliferation, migration, invasion, and promoted apoptosis. Knockdown of *MED19* decreased *p-AKT* and *p-mTOR* protein expression. Additionally, the downstream effectors of the *AKT/mTOR* pathway, *p70S6K1* and *4EBP1*, were affected by *MED19*. Notably, *MED19* expression was positively correlated with the infiltration levels of B cells, CD4^+^ T cells, CD8^+^ T cells, macrophages, *etc.*

**Conclusion:**

*MED19* is significantly upregulated in HCC tissues and cells. *MED19* may promote the progression of HCC *in vitro* and may be related to immune infiltration. Together, our data show that *MED19* could be considered as a new possible biomarker as well as a novel therapeutic target for HCC.

## Introduction

Hepatocellular carcinoma (HCC) is the most common primary malignancy of hepatocytes and serves as the third leading cause of cancer-related death worldwide (1).The treatment of HCC is limited. At present, surgery is the main treatment strategy for early HCC; however, following surgery recurrence and metastasis are common. Patients with advanced HCC are prescribed sorafenib, a specific molecular targeting drug, but drug resistance and side effects have become serious problems (2, 3). Furthermore, the prognosis of HCC is very poor with the 5-year survival rate reported at less than 5% (4). Therefore, researchers and clinicians must elucidate the molecular mechanisms of the occurrence, development, invasion, and metastasis of HCC to find and develop new therapeutic targets.

Mediator is an evolutionarily conserved multi-protein complex (5, 6). As an important part of the transcription mechanism of eukaryotes, the Mediator complex participates in gene expression and mediates the interaction between different proteins (7–9). In humans, mutations, or changes in the Mediator complex have a wide-ranging impact on the occurrence and development of a variety of diseases including cancer.

In 2003, *MED19*, or lung cancer metastasis-associated protein 1, was cloned from lung large carcinoma and found to be an important part of the Mediator complex (10–12). *MED19* is mainly confined to the nuclear region of the cell (https://www.proteinatlas.org/ENSG00000156603-MED19/cell). Further, several studies have shown that *MED19* plays a key role in malignant tumor growth by regulating signals related to cell growth, differentiation, cell cycle, and apoptosis (13–16). Zhang et al. found that the expression of *MED19* in breast cancer tissues was significantly higher than that in adjacent tissues (17, 18). Further, *MED19* promoted breast cancer cell proliferation through the *EGFR/MEK/ERK* signaling pathway (19). In addition, studies have reported that the expression of *MED19* was positively correlated with the expression of bone morphogenetic protein 2 (*BMP2*) in bladder urothelial carcinoma bone metastasis and invasion (20). *MED19* transcription can activate the expression of endogenous *Tspan8*, and regulate the adhesion and invasion of melanoma in a *Tspan8*-dependent manner (21). As a member of the Mediator complex, *MED19* plays a key role in the activation and inhibition of tumor signal transduction and transcriptional regulation and has a role in the induction of other developmental diseases. Zou et al. (2011) reported that the inhibition of *MED19* reduced HCC cellular proliferation, induced cell-cycle arrest, and suppressed tumor formation (22). However, the specific mechanism explaining how *MED19* affects the occurrence and development of HCC is unclear. Therefore, an in-depth understanding of the biological function of *MED19* and its mechanism of action in HCC might be helpful in the identification of potential targets for clinical treatment.

In the present study, we show that *MED19* is upregulated in HCC tissues and that *MED19* upregulation was closely correlated with a poor prognosis. Furthermore, *MED19* knockdown was observed to modulate the migration, invasion, and apoptosis of HCC cells, and may promote the occurrence and development of HCC through the *AKT/mTOR* pathway. Additionally, *MED19* was correlated with tumor immune infiltration. Together, this study shows that *MED19* plays an important oncogenic role in the occurrence and development of HCC, and could be considered as a newpossible biomarker as well as a novel therapeutic target for HCC.

## Materials and Methods

### Public Datasets Analysis

Tumor Immune Estimation Resource (TIMER) 2.0 is a comprehensive resource for the systematic assessment of diverse cancer types. Using the TCGA database, TIMER 2.0 explores the differential gene expression between tumors and normal tissues, the correlation between gene expression and clinical results, and the analysis of tumor immune infiltration (23). Gene Expression Profiling Interactive Analysis (GEPIA) is a web server based on the visual analysis of the TCGA database (24). It provides several key interactive customization features, such as differential expression analysis, patient survival analysis, and related gene detection, *etc.* UALCAN is a comprehensive, user-friendly, and interactive web resource for analyzing cancer data (25). LinkedOmics is a platform that can access, analyze and compare cancer multi-omics data within and across tumor types (26). In the current study, the TIMER 2.0 database was used to analyze immune infiltration and the expression profile of *MED19* in different types of human cancers. Additionally, using the GEPIA datasets, the survival analysis of *MED19* in HCC was evaluated. Using the UALCAN dataset, factors related to *MED19* transcription in HCC were investigated. Using the LinkedOmics datasets, genes in HCC that are related to *MED19* were assessed and used to draw heat maps. Finally, the relationship between *MED19* and several key HCC regulators, including *AKT* and *mTOR* was assessed.

### Immunohistochemistry

Human HCC tissue specimens and adjacent non-tumor tissues were obtained from patients who underwent surgical hepatectomy. All patients were sourced from the Second Affiliated Hospital of Guilin Medical University, China and signed informed consent. Immunohistochemical staining analyses were performed using formalin-fixed paraffin-embedded tissue sections. The sections were deparaffinized, rehydrated, and incubated in EDTA at 120°C for 5 minutes for antigen repair. After incubating with 3% H2O2 at room temperature for 15 minutes, the sections were sealed with fetal bovine serum and incubated with primary antibody overnight. Goat anti-rabbit antibody coupled with HRP was used for immune detection. Finally, the immune complex was displayed with chromogenic substrate, and the sections were re-stained with hematoxylin. In order to reduce the non-specific binding of antibody, titration was used to optimize the concentration. The diagnosis of the liver cancer samples was verified by pathologists.

### Cell Lines and Cell Culture

The human hepatocellular carcinoma cell lines HepG2 and Huh7 are generated by our laboratory. The HepG2 and Huh7 cells were maintained in DMEM high glucose medium (Gibco; Thermo Fisher Scientific) containing 10% serum (South American Fetal Bovine, EXCELL).

### Plasmid Construction and Cell Transfection

The *MED19* knockdown plasmid was designed by Genechem, Shanghai, China. The sh-*MED19* sequence was as follows: sense 5’-CAGTACTCTTTCAATCCTAT-3’, irrelevant nucleotides not targeting any annotated human genes were used as the negative control (sh-NC): sense 5’-TTCTCCGAACGTGTCACGT-3’. Cell transfection with plasmids was conducted using Lipofectamine 2000 (Invitrogen, USA) in accordance with the manufacturer’s instructions.

### Cell Proliferation Analysis

Cell proliferation was measured by the MTT assay (Solarbio, China). Briefly, the cells were transfected with the sh-*MED19* plasmid or corresponding negative control and were then seeded into 96- well plates (5×10^3^ cells/well), and cultured at 37°C for 0 h, 24 h, 48 h, 72 h. After incubation, 20 ul of the MTT solution was added to each well and incubated for 4 h at each time point at 37°C. The MTT solution was aspirated and 200ul DMSO (Solarbio, China) was added to each well to dissolve the formazan crystals. Cellular proliferation in each well was quantified by measuring optical density using an EPOCH 2 microplate spectrophotometer at a wavelength of 490 nm. The transfected cells were used for cell colony formation assay. Approximately, 100-1000 cells were added per well in a six-well plate. The colonies were then fixed with 4% paraformaldehyde, stained with crystal violet solution and counted.

### Cell Migration and Invasion Assay

Cell migratory ability was assessed using the scratch assay. After transfection with the targeted plasmid, 10^6^ cells were seeded in a six-well plate with DMEM containing 2% serum. After adhering to the well, the cells were scratched with a 10 ul pipette tip. The migration distance was measured at 0 h, 24 h and 48 h, and the migration capacity was calculated. Cell invasion was measured in a 24-well plate and transwell chamber covered with Matrigel (CORNING, USA). The transfected cells were resuspended in serum-free media and counted. The upper chamber was inoculated with 10^5^ cells per well, and 10% serum media was added to the lower chamber of the 24-well plate as an inducer to trigger cell invasion. After 36 h, the bottom of the chamber was fixed with 4% paraformaldehyde, stained with 1% crystal violet and counted (100X).

### Cell Apoptosis Assay

After cell transfection for 24 h, the 6-well plate was redigested to observe the transfection efficiency. A sterile cover glass was placed in the 6-well plate in advance. The cells were then added to the 6 well-plate and cultured overnight to a density of approximately 50-80%. Following the culture period, the cells were fixed with 0.5 ml fixative solution for 10 min, stained with 0.5 ml Hoechst 33258 staining solution for 5 min, washed with anti-fluorescence quenching solution, and observed under a fluorescence microscope. Normal cell nuclei appeared blue, apoptotic nuclei were densely stained, or fragmented and densely stained, and whitish.

### Flow Cytometry for Apoptosis Detection

After 24 h of cell transfection to observe the transfection efficiency, apoptosis was measured using the Annexin V-Phycoerytirin (Annexin V-PE) cell apoptosis detection kit (C1065, Beyotime Biotechnology, China). These data were acquired by flow cytometry (Thermo Fisher, USA) and analyzed by Flowjo software.

### qRT-PCR

Total RNA was extracted using the TRIZOL. RNA was reverse transcribed to cDNA using the qRT-PCR kit (Thermo Scientific, USA) according to the manufacturer’s protocol. The PCR cycling conditions were as follows: 40 cycles with pre-denaturation at 95°C for 15 min, denaturation at 95°C for 10 s, and annealing and extension at 60°C for 32 s.

The primers used in this study were synthesized by Wuhan Genecreate Biological Engineering Co., Ltd, and were as follows: *MED19*-F: CTGACAGGCAGCACGAATCT, *MED19*-R: CTCCTTCACCTTCTTCCCACA; *AKT*-F: TACTCTTTCCAGACCCACGAC, *AKT*-R: AGGTTCTCCAGCTTGAGGTC; *mTOR*-F: CGCTGTCATCCCTTTATCGAC, *mTOR*-R: CAGAGTCAAGTGGTCATAGTCCG; *4EBP1*-F: CTCACCTGTACCAAAACACC, *4EBP1*-R: CCCGCTTATCTTCTGGGCTA; *p70S6K1*-F: GTGCTGTGGATTGGTGGAGT, *p70S6K1*-R: GAGGTAGGGAGGCAAATTGAG; *GAPDH*-F: AGAAGGCTGGGGCTCATTTG, *GAPDH*-R: AGGGGCCATCCACAGTCTTC. All samples were normalized to internal controls and fold changes were calculated based on relative quantification (2^-ΔΔCt^).

### Western Blot

48 h after cell transfection, the protein samples were extracted with high-efficiency RIPA cell lysis buffer and protease inhibitor (100:1). Then, the protein samples were electrophoresed on 10% sodium dodecyl sulfate-polyacrylamide (SDS-PAGE) gel, then transferred to polyvinylidene fluoride (PVDF) membrane and blocked with 5% skimmed milk powder for 2 h. The samples were incubated with the primary antibody overnight. All antibodies used in this study are shown in **Table 1**. The membrane was washed three times, incubated with the corresponding enzyme-labeled secondary antibodies for 1 h, including horseradish peroxidase (HRP) – conjugated anti-rabbit (cat. no. 111-035-003, 1:10000; Jackson ImmunoResearch, USA), and horseradish peroxidase (HRP) – conjugated anti-mouse (cat. no. 115-035-003, 1:10000; Jackson ImmunoResearch, USA). The signals of the protein bands were analyzed using ChemiDoc XRS+ biomolecular imaging system (BIO-RAD, USA). Analysis using Image J software.

**Table 1:** Western blot antibodies.

Antigens	Molecular Weight	Manufacturers	Application
**MED19**	36kDa	ab251866, Abcam, England	1:50 for IHC, 1:200 for WB
**AKT1**	55kDa	A11016, Abclonal, China	1:500 for WB
**p-AKT1-s473**	55 kDa	AP0098, Abclonal, China	1:500 for WB
**mTOR**	289 kDa	A2445, Abclonal, China	1:500 for WB
**p-mTOR(59.Ser2448)**	220 kDa	sc-293133, Santa Cruz, USA	1:200 for WB
**eIF4EBP1**	18kDa	A19045	1:500 for WB
**p-EIF4EBP1-S65**	18kDa	AP0032	1:500 for WB
**p70S6K1**	68kDa	A4898	1:500 for WB
**p-p70S6K1**	68kDa	AP0482	1:500 for WB
**LC3BI/II**	14/16 kDa	A19665, Abclonal, China	1:500 for WB
**β-actin**	43 kDa	BF0198, Affinity Biosciences, USA	1:5000 for WB
**GAPDH**	37 kDa	AF7021, Affinity Biosciences, USA	1:3000 for WB

### Statistical Analysis

All *in vitro* experiments were performed in triplicate. All statistical calculations and analyses were performed using GraphPad Prism 8.0.2 software (GraphPad Software, San Diego, CA, USA). The data were presented as mean ± SD. The comparison between the two groups was performed using by unpaired Student’s *t* -test (for parametric data). *P* values <0.05 were considered statistically different.

## Results

### *MED19* Was Up-Regulated in Hepatocellular Carcinoma

**Figure 1:**
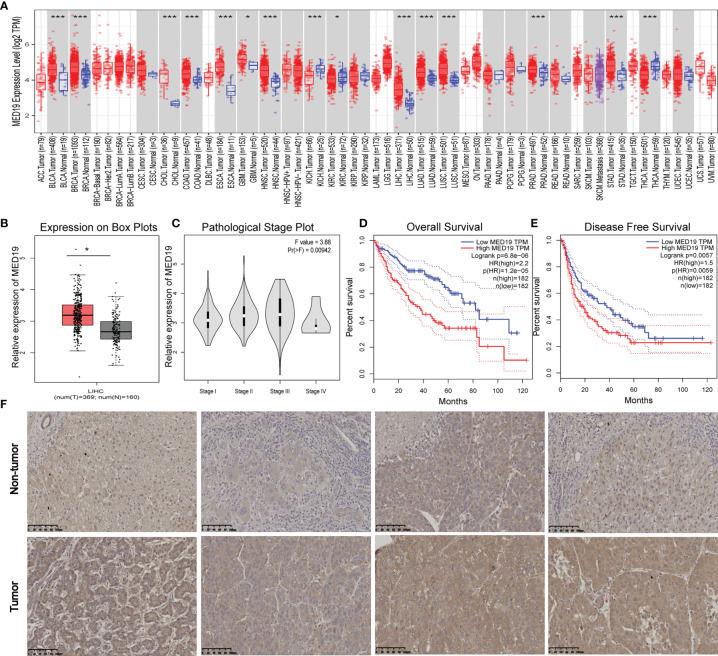
*MED19* is highly expressed in HCC. **(A)** Expression of *MED19* gene in different cancers or specific cancer subtypes. **(B)** *MED19* was upregulated in LIHC samples compared with normal tissues. **(C)** Based on the TCGA data, the expression of the *MED19* at different pathological stages of LIHC. **(D, E)** The higher expression of *MED19* was associated with a shorter overall survival time and disease-free survival time of HCC. **(F)** IHC images of HCC tissues showed that the expression of *MED19* in HCC tissues was higher than that in adjacent non-tumor tissues. *P < 0.05; ***P < 0.001.

**Figure 2:**
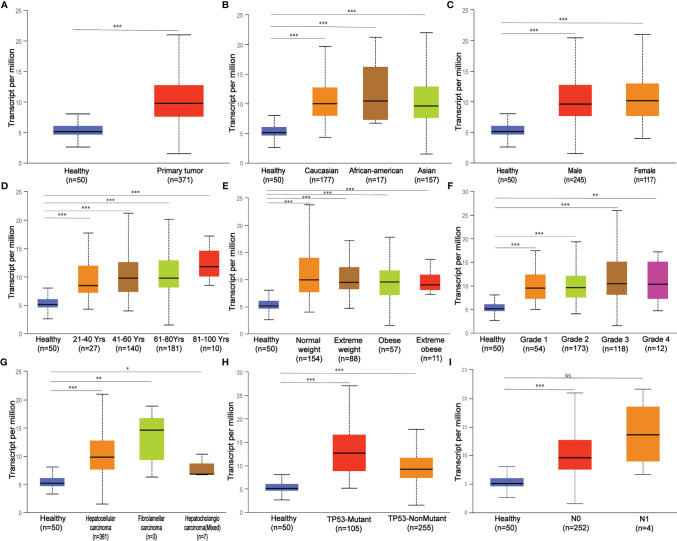
MED19 transcription in subgroups of patients with hepatocellular carcinoma stratified based on gender, age, and other criteria. **(A)** Boxplot showing relative expression of *MED19* in healthy and LIHC samples. **(B)** Boxplot showing relative expression of *MED19* in healthy individuals of any ethnicity or LIHC patients of Caucasian, African-American or Asian ethnicity. **(C)** Boxplot showing relative expression of *MED19* in healthy individuals of either gender or male or female LIHC patients. **(D)** Boxplot showing relative expression of *MED19* in healthy individuals of any age or LIHC patients aged 21-40, 40-60, 61-80, or 81-100 yr. **(E)** Boxplot showing relative expression of *MED19* in healthy individuals of any weight or LIHC patients weighted normal, extreme, obese, or extreme obese. **(F)** Boxplot showing relative expression of *MED19* in healthy individuals or LIHC patients with grade 1, 2, 3 or 4 tumors. **(G)** Boxplot showing relative expression of *MED19* in healthy individuals or LIHC patients of hepatocellular carcinoma, fibrolamellar carcinoma, or hepatocholangio carcinoma (mixed). **(H)** Boxplot showing relative expression of *MED19* in healthy individuals or LIHC patients with *TP53*-mutant or *TP53*- nonmutant. **(I)** Boxplot showing relative expression of *MED19* in healthy individuals or LIHC patients with no regional lymph node metastasis or metastases in 1 to 3 axillary lymph nodes. *P < 0.05; **P < 0.01; ***P < 0.001; NS, nonsignificant.

In this study, the carcinogenic effect of *MED19* during HCC was investigated. First, we analyzed the expression patterns of *MED19* in different tumor and non-tumor tissues. Using TIMER 2.0, *MED19* was shown to be highly expressed for bladder urothelial carcinoma (BLCA), breast cancer (BRCA), liver hepatocellular carcinoma (LIHC), lung adenocarcinoma (LUAD) and other types in the TCGA project (**Figure 1A**). Based on the GEPIA dataset, matching TCGA normal and GTEx data, *MED19* expression was found to be significantly higher in HCC tissues relative to other non-tumor tissues (**Figure 1B**). Based on the TCGA dataset, *MED19* expression was highest in stage III HCC tissues (**Figure 1C**). The high expression of *MED19* was associated with overall survival and disease-free survival, suggesting a poor prognosis (**Figures 1D, E**). In addition, within the clinical specimens collected, *MED19* was highly expressed in cancer tissues compared with paracancerous tissues (**Figure 1F**). The level of *MED19* transcription was significantly higher in HCC patients relative to healthy subjects in the subgroup analysis based on gender, age, race, tumor grade, *etc.* (**Figure 2**). Therefore, the expression of *MED19* may serve as a potential diagnostic indicator in HCC.

### *MED19* Knockdown Inhibited Proliferation and Promoted Apoptosis of HCC Cell

**Figure 3:**
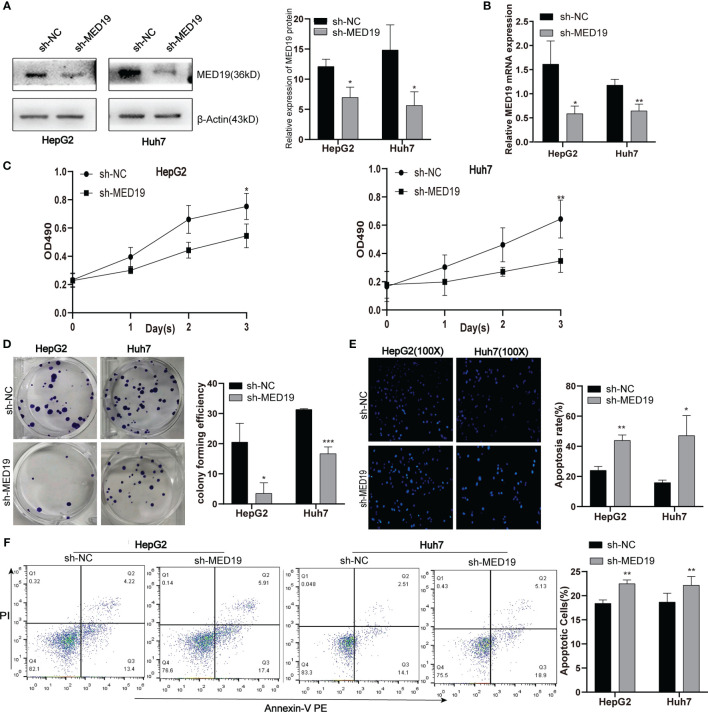
*MED19* knockdown inhibited HCC cell proliferation and promoted cell apoptosis. **(A, B)** Relative *MED19* RNA and protein levels in HepG2 and Huh7 cells after transfection with sh-*MED19* or sh-NC plasmid. **(C)** The viability of HepG2 and Huh7 cells in the sh-*MED19* and sh-NC group. **(D)** Influence of *MED19* knockdown on clone formation of HepG2 and Huh7 cells. **(E)** The apoptosis situation of HepG2 and Huh7 cells induced by Hoechst 33258. **(F)** The apoptosis situation of HepG2 and Huh7 cells by flow cytometry. *P < 0.05; **P < 0.01; ***P < 0.001. Perform three independent replicates. Statistical analysis is performed using an unpaired *t -*test. All results are expressed as mean ± SD.

In the current study, we used HepG2 and Huh7 cells as knockdown models to explore the potential biological function of *MED19*. shRNA was transfected into cells within a plasmid vector. The transfection efficiency was evaluated by fluorescence microscope and knockdown efficiency was evaluated by western blot and qRT-PCR (**Figures 3A, B**). The proliferation ability of Huh7 and HepG2 cells was detected by the MTT and colony formation assays. The downregulation of *MED19* significantly inhibited cell proliferation (**Figure 3C**) and colony formation efficiency (**Figure 3D**). Due to a higher transfection efficiency of HepG2 relative to Huh7, fewer HepG2 colonies were observed. In addition, we found that the apoptotic ability of HCC cells increased significantly after *MED19* was knockdown by Hoechst and flow cytometry (**Figures 3E, F**).

### *MED19* Knockdown Inhibited HCC Cell Migration and Invasion

**Figure 4:**
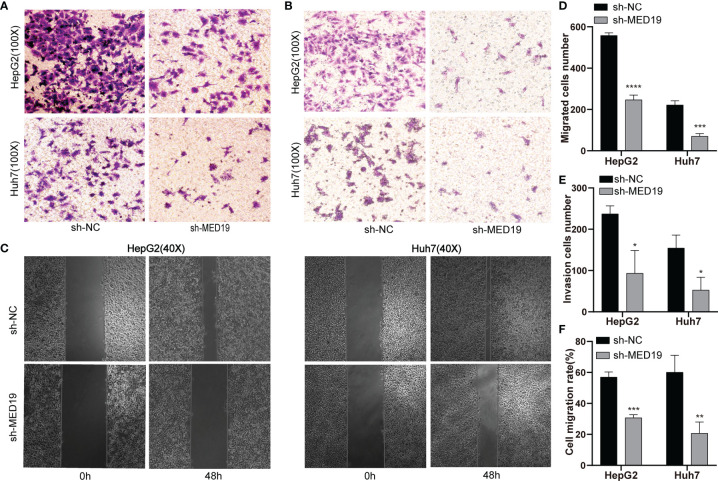
*MED19* knockdown inhibited the migration and invasion of HCC cells *in vitro*. **(A, D)** Transwell assay was used to detect the migration of HCC cells between the sh-NC and the sh-*MED19* group. **(B, E)** Transwell assay was used to detect the invasion of HCC cells between the sh-NC and sh-*MED19* groups. **(C, F)** Wound-healing was utilized to evaluate the effect of *MED19* knockdown on the migration of HCC cells. *P < 0.05; **P < 0.01; ***P < 0.001, ****P < 0.0001. Perform three independent replicates. Statistical analysis is performed using an unpaired *t -*test. All results are expressed as mean ± SD.

To explore the effect of *MED19* on cell migration and invasion, a transwell experiment was performed. The number of cells that crossed the chamber in the sh-*MED19* group was significantly lower than the sh-NC group (**Figures 4A, D**). The invasion experiment was performed in a chamber containing Matrigel. The knockdown of *MED19* inhibited the invasion ability of HepG2 and Huh7 cells (**Figures 4B, E**). Wound-healing experiments further confirmed the influence of *MED19* expression on the migration ability of HCC cells. The migration rate of HCC cells in the sh-*MED19* group was markedly lower than that in the sh-NC group (**Figures 4C, F**). Together, these data suggested that *MED19* may act as an oncogene and promotes migration and invasion in HCC.

### *MED19* Knockdown Inhibited the Activation of the *AKT/mTOR* Signaling Pathway *In Vitro*

**Figure 5:**
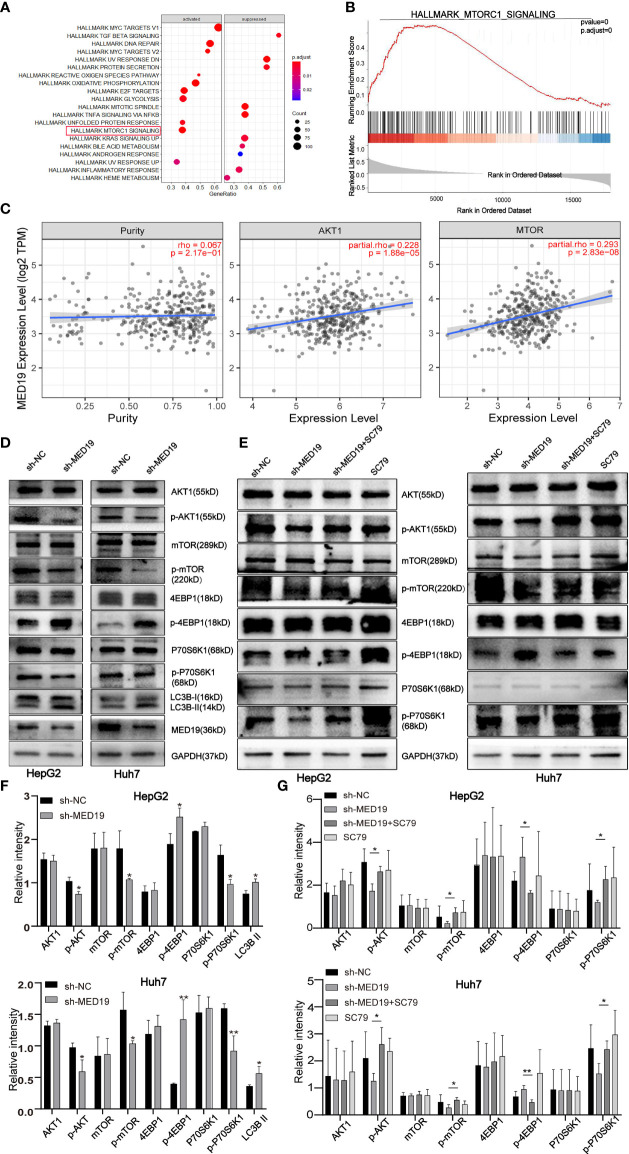
*MED19* knockdown inhibited *AKT/mTOR* signaling pathway. **(A, B)** Pathway enrichment analysis of *MED19* in HCC. **(C)** Co-expression analysis of *MED19* and *AKT/mTOR* pathway genes. **(D, F)** The protein expression of *AKT*, *p-AKT*, *mTOR*, *p-mTOR*, *p70S6K1, p-p70S6K1, 4EBP1, p-4EBP1*, and *LC3B I/II* in HepG2 and Huh7 cells from the sh-*MED19* and sh-NC groups. **(E, G)** SC79 enhances the expression of *p-AKT, p-mTOR*, and *p-p70S6K1* but inhibits the expression of *p-4EBP1*. *P < 0.05; **P < 0.01. Perform three independent replicates. Statistical analysis is performed using an unpaired *t -*test. All results are expressed as mean ± SD.

To further explore the functional role of *MED19* in cell proliferation, migration, invasion, and apoptosis, we aimed to identify the potential mechanism of *MED19* in HCC cells. Initially, data from the TCGA database indicated that *MED19* was positively correlated with *AKT* and *mTOR* (**Figures 5A-C**). To further explore the potential relationship between *MED19* and other genes related to the *AKT/mTOR* signaling pathway (*AKT, p-AKT, mTOR, p-mTOR, 4EBP1, p-4EBP1, p70S6K1, p-p70S6K1*), proteins expression was determined by western blot. Western blot analysis revealed decreased *p-AKT, p-mTOR, p-p70S6K1* protein expression, and increased *p-4EBP1* expression in *MED19*-downregulated cells (**Figures 5D, F**). Together, these data indicate that the activity of the *AKT/mTOR* pathway was decreased in *MED19*-depleted HCC cells. However, after treatment with noval AKT activator, SC79, the activation of the *AKT/mTOR* signaling pathway in HCC was partially restored (**Figures 5E, G**). This suggests that SC79 increased the phosphorylation of *AKT* and *mTOR* in *MED19* knocked down HCC cells, thus further supporting the hypothesis that the *AKT/mTOR* pathway is the molecular target of *MED19* in HCC cells.

**Figure 6:**
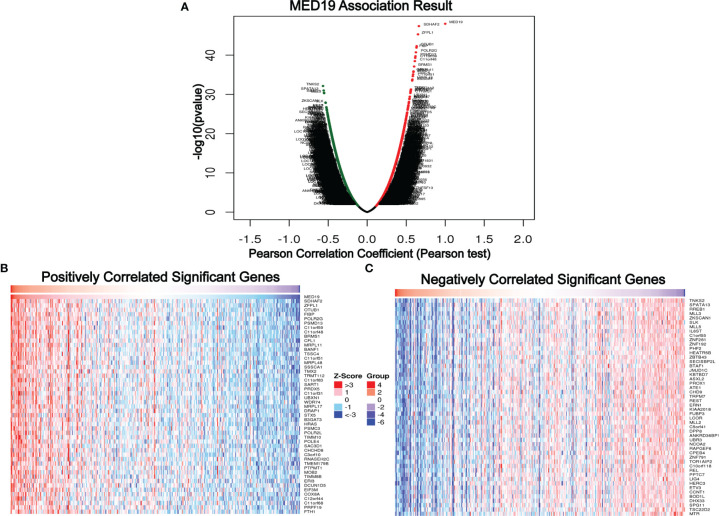
Genes differentially expressed in correlation with *MED19* in hepatocellular carcinoma. **(A)** A Pearson test was used to analyze correlations between *MED19* and genes differentially expressed in HCC. In the volcano map, red indicates positively correlated genes and green indicates negatively correlated genes. **(B, C)** Heat maps showing genes positively and negatively correlated with *MED19* in HCC (TOP 50).

Previous studies have reported that abnormal *AKT/mTOR* signals were closely related to autophagy (27, 28). Based on this, we speculate that *MED19* may reduce autophagy in HCC cells. Therefore, WB was used to determine the expression level of key autophagy-related proteins, *LC3B-I* and *LC3B-II*. The protein level of *LC3B-II* in the *MED19* knockdown group was higher than the corresponding control group (**Figures 5D, F**). In general, the downregulation of *MED19* inhibited the proliferation, migration, invasion, and apoptosis of HCC cells through the *AKT/mTOR* signaling pathway and may be related to autophagy. In addition, using the LinkedOmics database, we found that *MED19* in HCC was related to many genes. In **Figure 6**, we highlight genes that are positively and negatively related to *MED19* in HCC (**Figures 6A-C**).

### Correlation Between *MED19* Expression and Immune Cells Infiltration and Markers of Different Subsets in HCC

**Figure 7:**
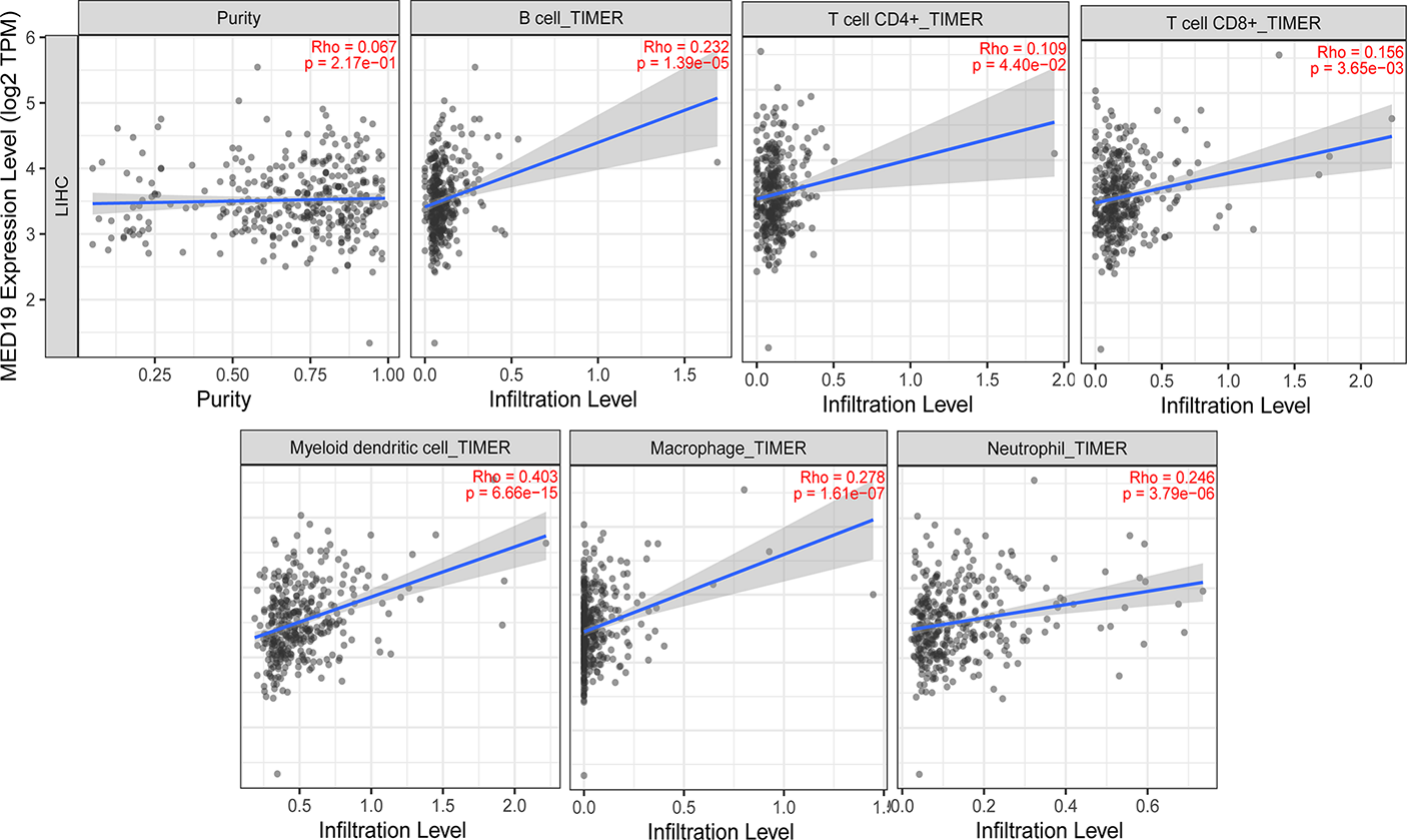
Correlation analysis between the expression of *MED19* and the level of immune cells infiltration in HCC. After tumor purity adjustment, *MED19* expression in HCC was positively correlated with infiltration levels of B cells, CD4^+^ T cells, CD8^+^ T cells, myeloid dendritic cells, macrophages, and neutrophils.

In the process of tumor invasion, immune cells, as an important part of the tumor microenvironment (TME), are closely related to the occurrence and development of cancer (29–31). Cancer immunotherapy utilizes engineered auto-immune cells to eliminate tumor cells. Therefore, understanding the infiltrating immune cells in the TME is essential for deciphering the mechanism of immunotherapy, defining predictive biomarkers, and identifying new therapeutic targets. It is reported that cancer-associated fibroblasts in the tumor microenvironment matrix are involved in regulating the function of various tumor-infiltrating immune cells (32, 33). Studies have found that the expression of tumor-related genes is related to the infiltration level of CD4^+^ T cells, CD8^+^ T cells, macrophages, *etc.* (29, 34–40). The immune cells analyzed in HCC tissues included CD8^+^ T cells, B cells, tumor-associated macrophages (TAMs), monocytes, M1 and M2 macrophages, neutrophils, and natural killer (NK) cells. To explore the role of *MED19* in immune responses within the HCC microenvironment, TIMER 2.0 was used to assess the potential relationship between the infiltration level of different immune cells and the expression of the *MED19* gene in HCC. Because tumor purity affects the analysis of immune cell infiltration, the correlation analysis of tumor purity has been adjusted. These results indicate that the expression of *MED19* in HCC was significantly correlated with the increased expression of marker genes in B cells, CD8^+^, CD4^+^, myeloid dendritic cells, macrophage, and neutrophils (**Figure 7**). Together, these data indicate that high *MED19* expression creates an immunosuppressive microenvironment supports HCC progression. Therefore, as a potential target of HCC, *MED19* may be beneficial for future immunotherapy.

## Discussion

*MED19* is crucial in stabilizing Mediator’s complex’s transcriptional regulation processes (41–43). Numerous studies have shown that *MED19* plays a role in tumor growth, migration, invasion, and apoptosis of various cancer types. In this study, we observed that the expression of *MED19* in HCC tissue was increased compared with normal liver tissue, which was confirmed by IHC.

The high expression of *MED19* in HCC patients indicates a poor prognosis, suggesting that the expression of *MED19* may play an important role in HCC metastasis. Cui *et al.* found that inhibiting the expression of *MED19* inhibited the proliferation and tumorigenesis of human prostate cancer cells (44, 45), and also inhibited tumor growth and metastasis in colorectal cancer (46). *MED19* knockdown inhibited the proliferation and migration of bladder cancer cells by down-regulating the *WNT/β-catenin* signaling pathway (47). Given these results, through a series of experiments, we found that knocking down *MED19* significantly reduced the ability of migration, invasion, proliferation, and colony-formation of HCC cells. In addition, *MED19* knockdown significantly increased the proportion of apoptotic HCC cells. Together, our data showed that *MED19* was related to the tumorigenesis and development of HCC, which may be a related oncogene of HCC.

Tumor immunotherapy based on immune infiltration is a current research hotspot and which still requires more investigation and optimization. Immune cells influence the tumor microenvironment and affect tumor progression and metastasis (18, 37, 48, 49). Based on many findings, we evaluated the correlation between the abnormal expression of *MED19* in HCC and immune infiltrating cells. The results showed that the expression of *MED19* was significantly correlated with the expression of B cells, CD8^+^, CD4^+^ and other cell-related genes.

Finally, based on the role of *MED19* in the proliferation, migration, and invasion of HCC, we explored the mechanism of *MED19* in the *AKT/mTOR* signaling pathway. The *AKT/mTOR* signaling pathway plays an active role in promoting tumor invasion and metastasis (50–53). Here, we show that *MED19* knockdown reduced the expression of *p-AKT*, *p-mTOR* and *p-P70S6K1*, but increased the expression of *p-4EBP1*. Additionally, SC79, an *AKT* agonist, partially restored the expression of *p-AKT*, *p-mTOR, p-p70S6K1*, and *p-4EBP1*. In summary, *MED19* knockdown inhibited the proliferation, migration, and invasion of HCC through the *AKT/mTOR* signaling pathway.

In obesity studies, adipose tissue inflammation is a key process that promotes cancer (54, 55). The tumor-promoting effect of obesity alters the level of the microenvironment and inflammatory Mediators and affects the level and function of immune infiltrating cells. Dean *et al.* found that *MED19* regulates adipogenesis and participates in the process of fat metabolism by mediating *PPAR-γ* (56). In addition, *MED19* binds to *GATA* transcription factors and regulates *GATA*-driven genes together with *MED1* (57).

Our study showed that *MED19* affects HCC oncogenesis through the *AKT/mTOR* pathway and may be related to autophagy. Based on this result, further experiments to determine other factors in this possible pathway are urgently needed. Primarily, these studies should aim to further determine the correlation between *MED19* and autophagy. Secondly, the effect of *MED19* supplementation on HCC cyclin protein *Cyclin D1/B1* and apoptosis protein *Bax, Bcl-2*, *etc.* should be further elucidated. Thirdly, *in vivo* tumor formation experiments should be elaborated upon and the effect of *MED19* supplementation on HCC should be well defined *in vivo* and *in vitro*. Lastly, future work should explore the role and mechanism of the *MED19*-gene(s) axis in HCC.

## Conclusion

In summary, the data provided in this article show that *MED19*, as an oncogene, plays an important role in the proliferation, migration, and invasion of HCC cells through the *AKT/mTOR* signaling pathway, and may be related to autophagy. Therefore, *MED19*, as a potential biomarker for HCC diagnosis, may represent a potential therapeutic target for HCC treatment. Further efforts and investigations are needed to clarify the tumor-promoting mechanism of *MED19*.

## Data Availability Statement

The original contributions presented in the study are included in the article/supplementary material. Further inquiries can be directed to the corresponding authors.

## Author Contributions

All authors participated in the design and performed of the experiments. YTZ wrote the article. YTZ and PQ contributed to the bioinformatics analysis. XX, ML, and HH analyzed the data. YLZ and JY directed the study and modified the article.

## Funding

This work was supported by the National Natural Science Foundation of China [NSFC, No. 82160517], the Scientific Research and Technology Development Program of Guangxi [grant number AD18281009, AD18281010], and Thousands of Young and Middle-aged Backbone Teachers in Guangxi colleges and Universities Training Plan.

## Conflict of Interest

The authors declare that the research was conducted in the absence of any commercial or financial relationships that could be construed as a potential conflict of interest.

## Publisher’s Note

All claims expressed in this article are solely those of the authors and do not necessarily represent those of their affiliated organizations, or those of the publisher, the editors and the reviewers. Any product that may be evaluated in this article, or claim that may be made by its manufacturer, is not guaranteed or endorsed by the publisher.
